# Rapid identification of COVID wastewater surges in the absence of case data

**DOI:** 10.1128/msphere.00652-25

**Published:** 2026-04-02

**Authors:** Victoria I. Verhoeve, Joshua Lambert, Amy Jones, Timothy Driscoll

**Affiliations:** 1 Department of Biology, West Virginia University 240297, Morgantown, West Virginia, USA; Kanazawa Daigaku, Kanazawa, Japan

**Keywords:** infectious disease, wastewater testing, SARS-CoV-2, bioinformatics

## Abstract

Genetic testing of community wastewater (wastewater surveillance) is a valuable tool for following trends in the abundance of SARS-CoV-2 and other infectious disease pathogens over time. Wastewater surveillance is increasingly important in the absence of corresponding epidemiological data, particularly for infectious diseases with limited timely data on clinical case incidences. Due to the inherent noise in wastewater data, a single sample is not sufficient to identify a sustained trend in the abundance of a target. This challenge is magnified in resource-limited settings where samples may be collected only once or twice per week. In this work, we collected 24-h composite samples of wastewater daily from a single facility for nearly 4 years. We use this high-frequency data set to describe a method for identifying trends in SARS-CoV-2 abundance in wastewater based on a variety of collection frequencies. Our results indicate that collecting two 24-h composites per week for 2 weeks is sufficient to accurately identify a SARS-CoV-2 surge. We conclude that low-frequency wastewater sampling performs reasonably well in identifying trends in a timely fashion.

**IMPORTANCE:**

Wastewater surveillance is increasingly being used to track trends in infectious disease targets such as SARS-CoV-2, often in the absence of widespread clinical data. In order to interpret the results of wastewater surveillance appropriately in these contexts, it is important to understand how to identify a surge in abundance in time to provoke appropriate downstream responses. This information can be used to adjust collection strategies to optimize target surveillance, particularly in resource-limited settings.

## INTRODUCTION

Wastewater testing for infectious diseases rose to prominence in the United States in 2020 at the onset of the COVID-19 pandemic, as an inexpensive method for tracking community-level trends in SARS-CoV-2, the virus that causes COVID-19 (1–4). Since that time, wastewater testing has gained widespread use as a method for following community trends in SARS-CoV-2 (5) and many other targets of public health concern, including influenza (6), norovirus (7), Mpox (8), poliovirus (9), measles (10, 11), and pertussis (12). Although wastewater testing cannot be used to identify infected individuals directly, it has been shown repeatedly that the abundance of a pathogen in wastewater aligns well with clinical case counts in the sewershed (12–16). In the case of infections with a significant pre-symptomatic or asymptomatic component, such as COVID-19 or measles, wastewater can serve as a crucial leading indicator for clinical cases and hospitalizations (3, 11, 17). In the absence of widespread individual testing, wastewater testing facilitates an ongoing assessment of infection risk that can be valuable in building resilient health care systems, particularly in low-resource settings (18–20).

Although the exact methods can vary (5), the general approach to wastewater testing involves collection of a wastewater sample, concentration of microbial particles, extraction of nucleic acids, and quantification of genetic target(s) of interest using a method such as quantitative polymerase chain reaction (PCR). Most workflows utilize composite sampling, wherein wastewater is collected over a 24-h period by combining small volumes of wastewater collected at regular intervals. Composites can be either time-based (e.g., collected for 5 min every 15 min for 24 h) or flow-based and are commonly collected using an automated sampling unit (autosampler).

Wastewater represents a community-level composite derived from a variety of inputs. The exact composition of a wastewater matrix varies over both geographic location and time and may, at any time, include input from private households, apartments, businesses, mass-gathering venues, community centers, health care facilities, and industry. In addition to human waste, wastewater may include detergents, grease, chemical runoff, industry and agricultural waste, material disposed of from hospitals, household waste, and input from wildlife. These components can affect the wastewater testing laboratory workflow at several steps; most notably, concentration of biological particles, extraction of nucleic acids, and PCR quantification (21). All of this complexity introduces significant noise in wastewater testing data and presents a formidable challenge for the accurate interpretation and comparison of testing results across time and location.

In September 2020, the United States Centers for Disease Control and Prevention (CDC) spearheaded the creation of the National Wastewater Surveillance System (NWSS), with the goal of building national capacity to track the distribution of the SARS-CoV-2 virus in community wastewater (5). NWSS provides crucial financial and logistical support for ongoing efforts by local, state, and tribal jurisdictions that engage in wastewater testing for infectious disease across their communities. Individual jurisdictions that participate in NWSS are organized under six wastewater Centers of Excellence (CoEs) that coordinate training, resource sharing, and communication among stakeholders, including public health departments, facility operators, and testing laboratories. Individual NWSS testing sites serve diverse populations across the country that range in size from a few dozen to a few million people, although facilities that serve larger populations are prioritized by most jurisdictions. Nearly 90% of all sites enrolled in the NWSS program are wastewater treatment facilities, with the remaining fraction comprised of universities and schools, healthcare facilities, correctional facilities, and upstream manhole locations (5).

NWSS encourages a collection frequency of two 24-h composite samples per week from each facility to ensure continuity of data on the NWSS dashboard. As of May 2025, this recommendation remains unchanged. Previous work has shown that a minimum of two samples weekly is required to maintain a good correlation between SARS-CoV-2 abundance in wastewater and clinical case counts of COVID (14). This work has demonstrated the usefulness of wastewater testing; however, it remains an open question how quickly one can identify a sustained change in a wastewater trend. This takes on additional importance in the absence of widespread individual testing, or for pathogens with significant asymptomatic or presymptomatic spread where clinical cases become even more of a lagging indicator of transmission. In the current study, we pose this question in a slightly different way: what collection frequency is sufficient to identify a sustained change in the wastewater trend? This information would improve our ability to interpret wastewater trends and advance our ability to respond effectively in the absence of widespread individual testing; furthermore, it would be valuable in informing resource distribution, particularly in low-resource settings.

In this study, we set out to determine how collection frequency affects the accurate identification of sustained SARS-CoV-2 increases (surges) in wastewater. Taking advantage of a long-term wastewater data set that comprised daily collections from a single treatment facility over nearly 4 consecutive years (2021–2024), we parameterized a model for identifying the start and end of surges in subsets of the data and tested the accuracy of the model against surges identified using the full data set. Our best-performing model used two 24-h composite samples collected twice per week. We conclude that collecting two 24-h composites per week can be used to identify surges in wastewater SARS-CoV-2 in a reasonable amount of time (10 days) to help inform effective public health action.

## RESULTS

### Wastewater and case counts align during widespread individual testing

**Fig 1:**
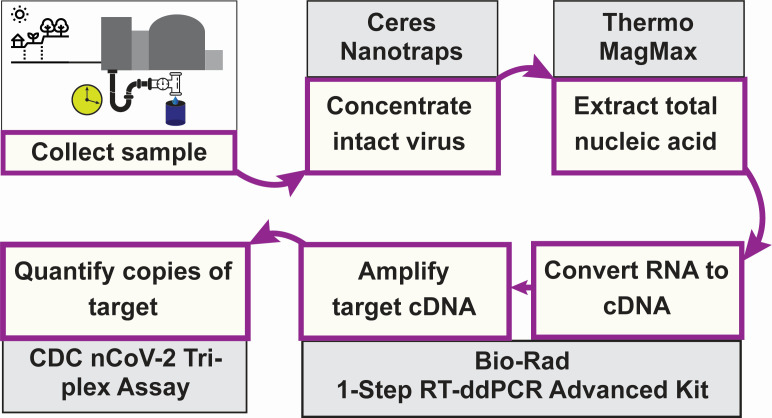
Overview of wastewater testing workflow used in the current study. Samples are collected as 24-h flow-based composites at the treatment facility, brought to the testing lab on ice, and processed within 24 h of receipt. Biologicals are concentrated from 10 mL of the liquid fraction and total nucleic acid extracted directly thereafter. SARS-CoV-2 abundance is quantified using ddPCR on a Bio-Rad QX600 with a triplex assay that simultaneously quantifies SARS-CoV-2 N1, SARS-CoV-2 N2, and human RNase P.

**Fig 2:**
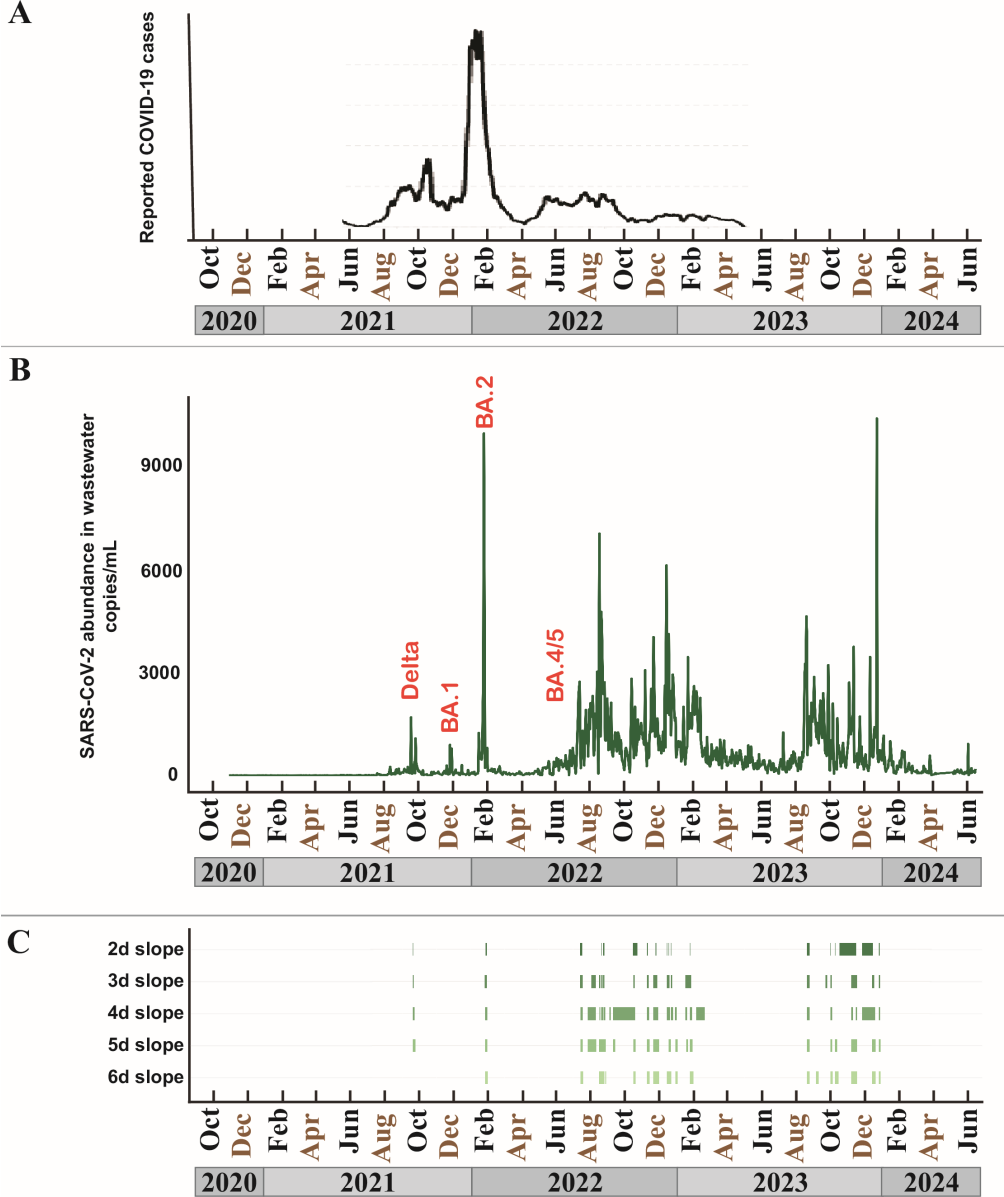
(**A**) Trends in daily new cases of COVID-19 for the county that contained the study facility, taken from covidactnow.org. (**B**) Daily SARS-CoV-2 abundance in copies per milliliter of raw wastewater at the study facility. Peaks in abundance that overlap with a dominant clinical variant of concern in the state are labeled in orange, where a single dominant variant could be identified (before August 2022). (**C**) Schematic of control surges identified using the full data set shown in (**B**). Each green block represents the start and end of a surge. Each row represents a different model using the slope over 2–6 day rolling windows. See Materials and Methods for details.

Our collection and assay workflow is shown in Fig. 1. This study was conducted over the period November 2020 to June 2024 using a multiplex RT-ddPCR assay that quantifies the SARS-CoV-2 N1 and N2 targets (2019-nCoV CDC droplet digital PCR triplex probe assay, Bio-Rad Laboratories). The abundance of these two targets in our study data is tightly correlated, with an *R*^2^ value of 0.9841 (Fig. S1); hence, we report just the N2 results here for clarity. As with previous studies, case counts (22) for the surrounding county (Fig. 2A) generally aligned with our wastewater data (Fig. 2B) when individual testing was still being widely reported. That includes increases in wastewater signal concomitant with the appearance of Delta, BA.1, BA.2, and BA.4/5 variants in clinical cases. In May of 2023, the federal COVID public health emergency expired and the CDC stopped collecting case data, leading to a decoupling of wastewater trends and case counts.

### Model construction and application

**Fig 3:**
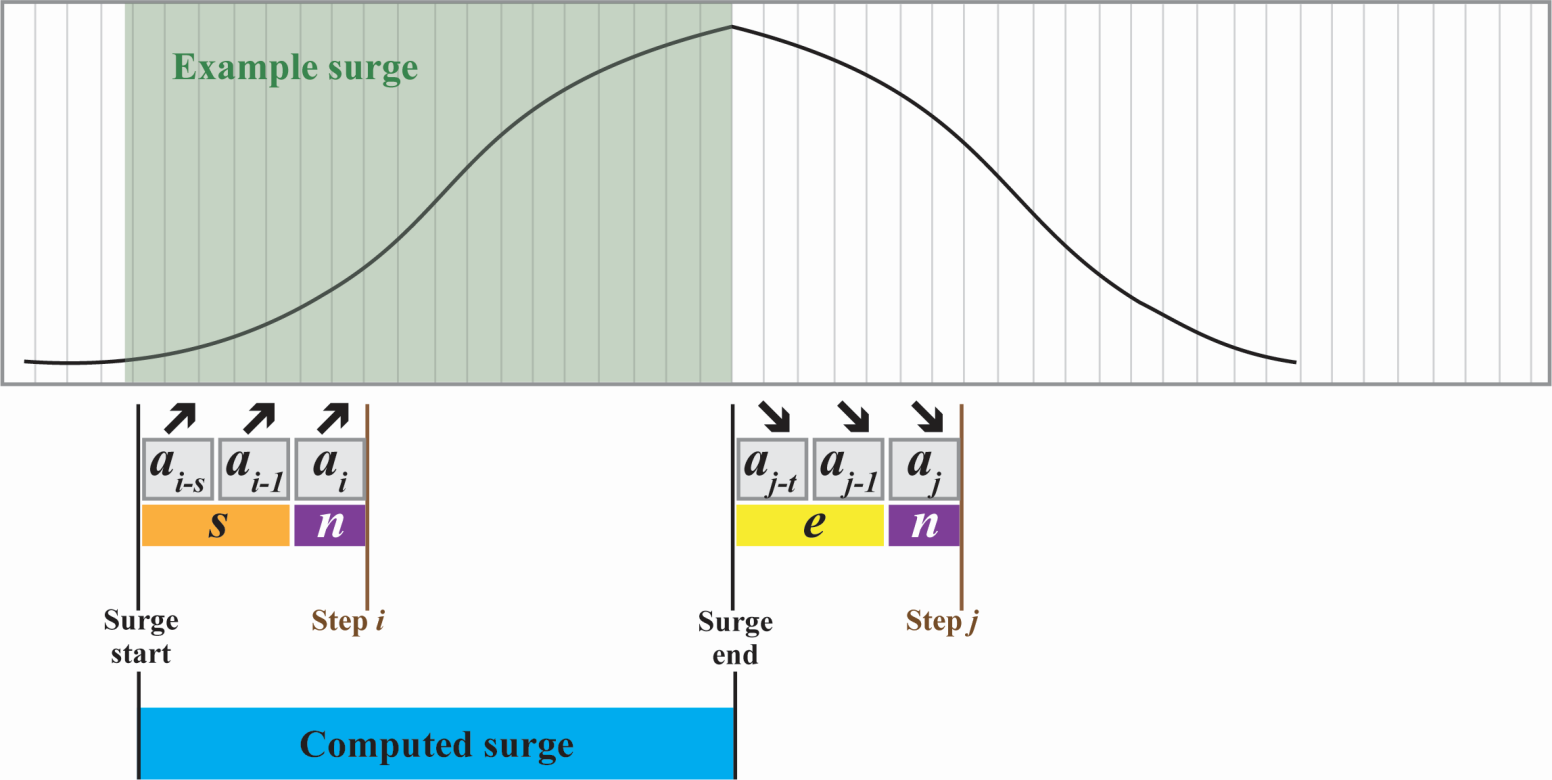
The start of a computed surge was defined as consecutive increases across *s* rolling windows of *n* samples. Similarly, the end of a computed surge was defined as consecutive decreases across *e* rolling windows of *n* samples. A surgeset was created by iterating in this manner across time. A set of 26,750 computed surgesets was created by iterating across subsets of the full data set (1–6 samples per week); parameterizing each subset over values of *n*, *s*, and *e*; and comparing against five different model (control) sets. See Materials and Methods for full details.

Figure 3 outlines our model construction, which functions by iterating through a set of abundance data ordered by collection date. There are four primary model parameters that can be customized: (i) *n*, the width of the rolling window of samples that comprises a model step; (ii) *s*, the number of consecutive steps to consider when detecting the start of a surge; (iii) *e*, the number of consecutive steps to consider when detecting the end of a surge; and (iv) *f*, the frequency of collection (samples per week) to include in the base abundance data set. Each combination of model parameters comes with an associated cost in days, *d*, required to identify a change in data trend. Each surge has a specific value of *d*, so the mean for all surges in a surgeset is reported as the value of *d* for the surgeset itself.

**Fig 4:**
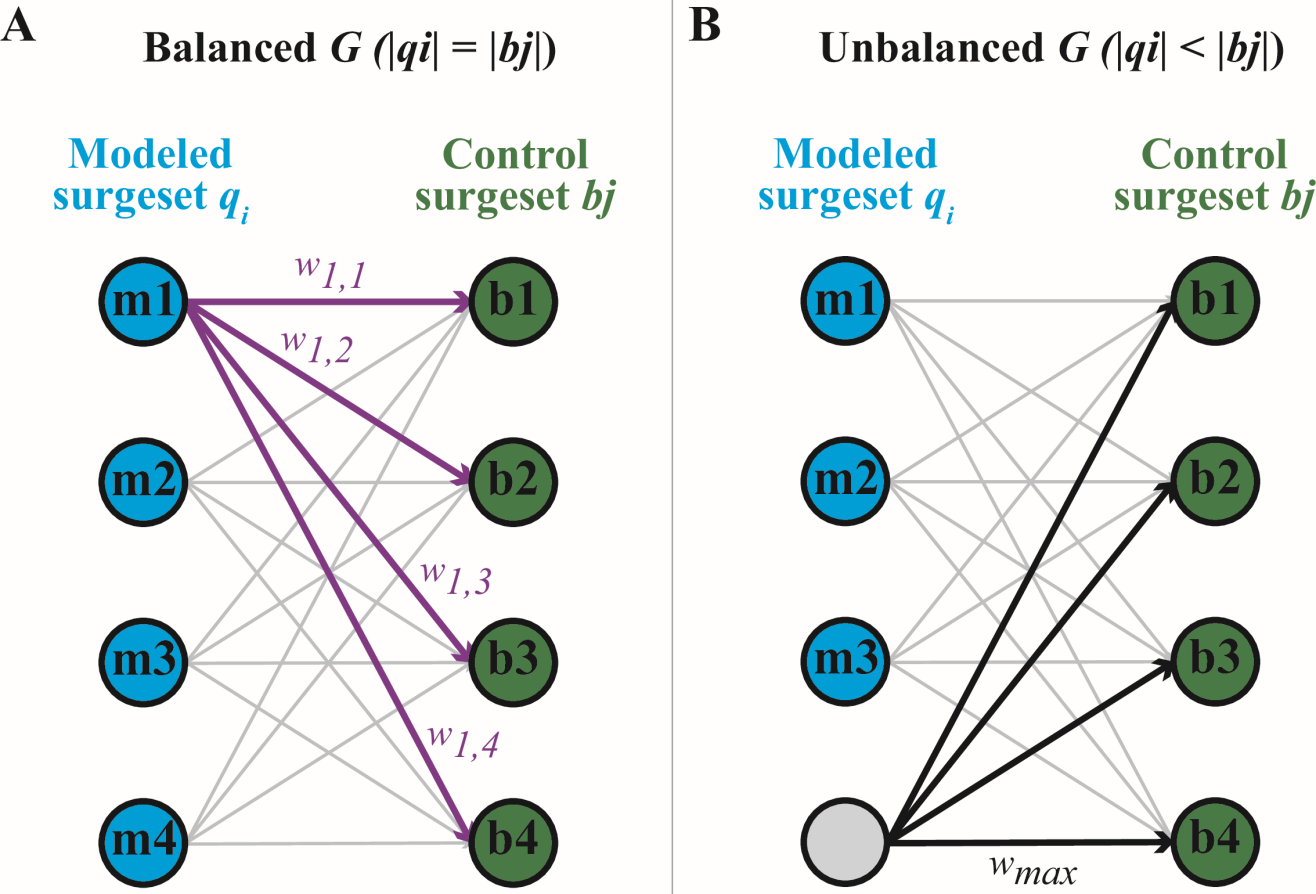
(**A**) In order to compare each modeled surgeset (blue) with each control (green), a bipartite weighted directed acyclic graph was created, with the surges as nodes. Edges joined every modeled surge to every control surge, making it a completely connected graph. Weights were calculated as the Euclidean distance between the component surges. (**B**) If the number of modeled and control surges was not equal, the DAG was balanced by adding “dummy” nodes (gray) to the smaller side of the graph. Each dummy node was connected as in (**A**), and the edges were weighted with the maximum weight between non-dummy nodes in the DAG. See Materials and Methods for full details.

Our iterative process generated a total of 26,750 surgesets, each consisting of pairs of dates corresponding to the start and end of the model surges. We scored each surgeset by comparing its date pairs to those of each control set constructed from the complete abundance data set. Our scoring approach (Fig. 4) utilized balanced directed acyclic graphs (DAGs), wherein the model and control surgesets being compared had to contain the same number of surges. To account for this requirement, “dummy” nodes were added to balance any unbalanced DAGs. Edges incident on a dummy node were assigned the largest weight found among the non-dummy nodes of the graph.

The complete set of model surgesets generated for this study can be found in Table S1. In addition, the study data (including all 140,479 computed surges) and our computational workflow are freely available via our GitHub repository, wwsurges (23). The codebase is a series of sequential R scripts that can be chained together using the included Bash control files. All of the parameters are tunable by the user, including the choice of dummy edge weights.

### Two samples per week are sufficient to identify most surges

**Fig 5:**
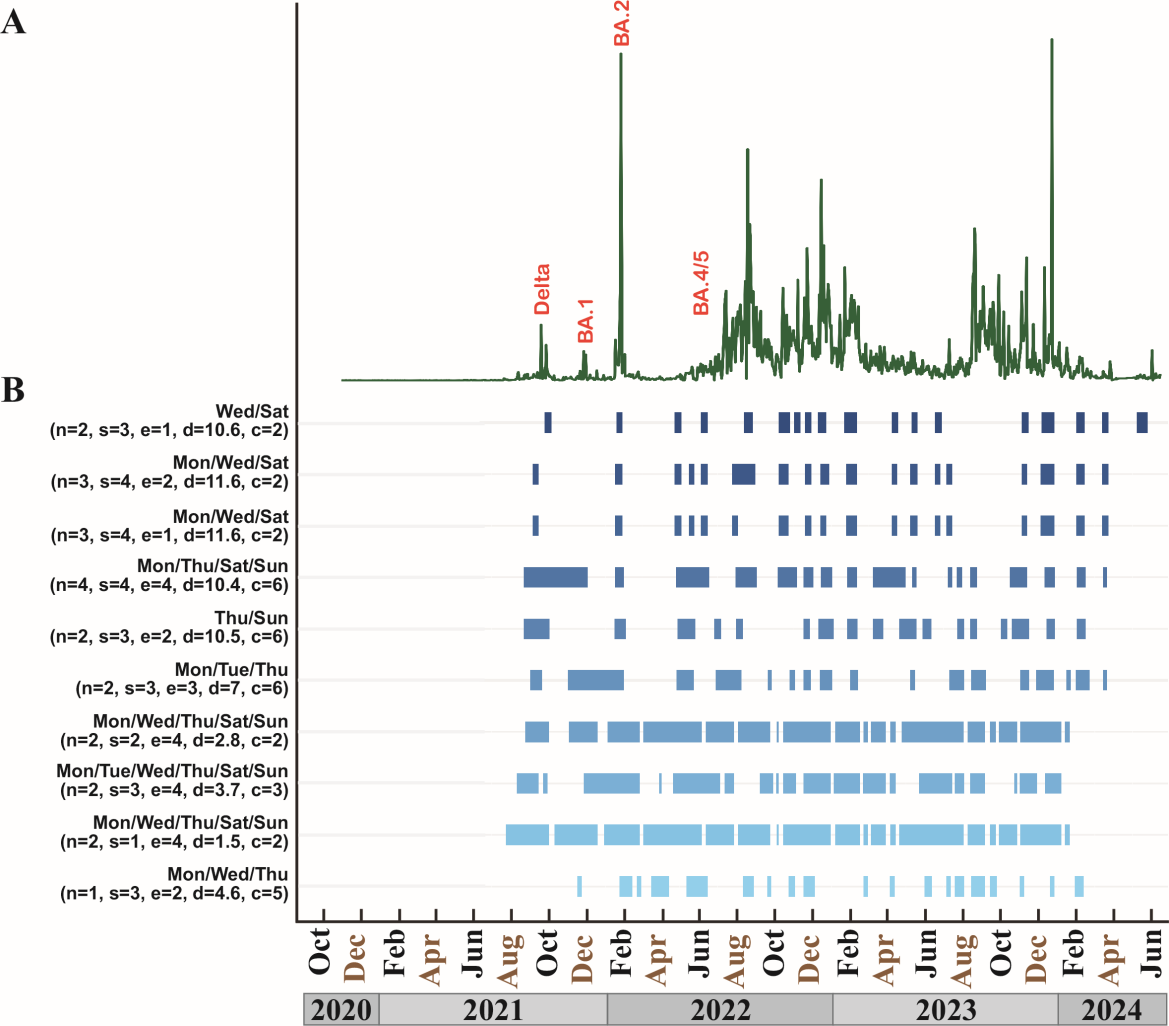
(**A**) Plot of daily SARS-CoV-2 abundance from the study facility (same as Fig. 2, provided here as a reference). (**B**) Top 10 scoring computed surgesets regardless of control model. For each computed set, *n* is the width of the sampling window; *s* is the number of consecutively increasing windows at the surge start; *e* is the number of consecutively decreasing windows at the surge exit; *d* is the calendar days required to identify a surge; and *c* is the control model used to score the surgeset. See Materials and Methods for full details.

Figure 5 shows the plot of daily abundance values (Fig. 5A) compared to the top 10 scoring model surgesets (Fig. 5B), with the top-scoring model at the top of the panel. It also shows the values for *n*, *s*, and *e* used to parameterize each model, the mean number of days necessary to identify the surges (*d*), and the control model used to score it (*c*). The top-scoring surgeset used two collections per week (2-day model), collected 3 days apart, and required a mean of 10.6 days to identify a surge. The pattern of surges in this top-scoring model aligned well with the complete set of abundance data, although there were differences. For example, the model did not identify the upward trend associated with the introduction of the BA.1 variant (December 2021), although it was not alone: only one of the top 10 models was able to identify the BA.1 surge accurately. The top-scoring model also missed the upward trend in abundance data starting in June 2022, only picking it up in August. It did not predict a surge at all during the August to October 2023 period, although the abundance data did show substantial activity.

### Three samples per week is the most flexible collection frequency

**Fig 6:**
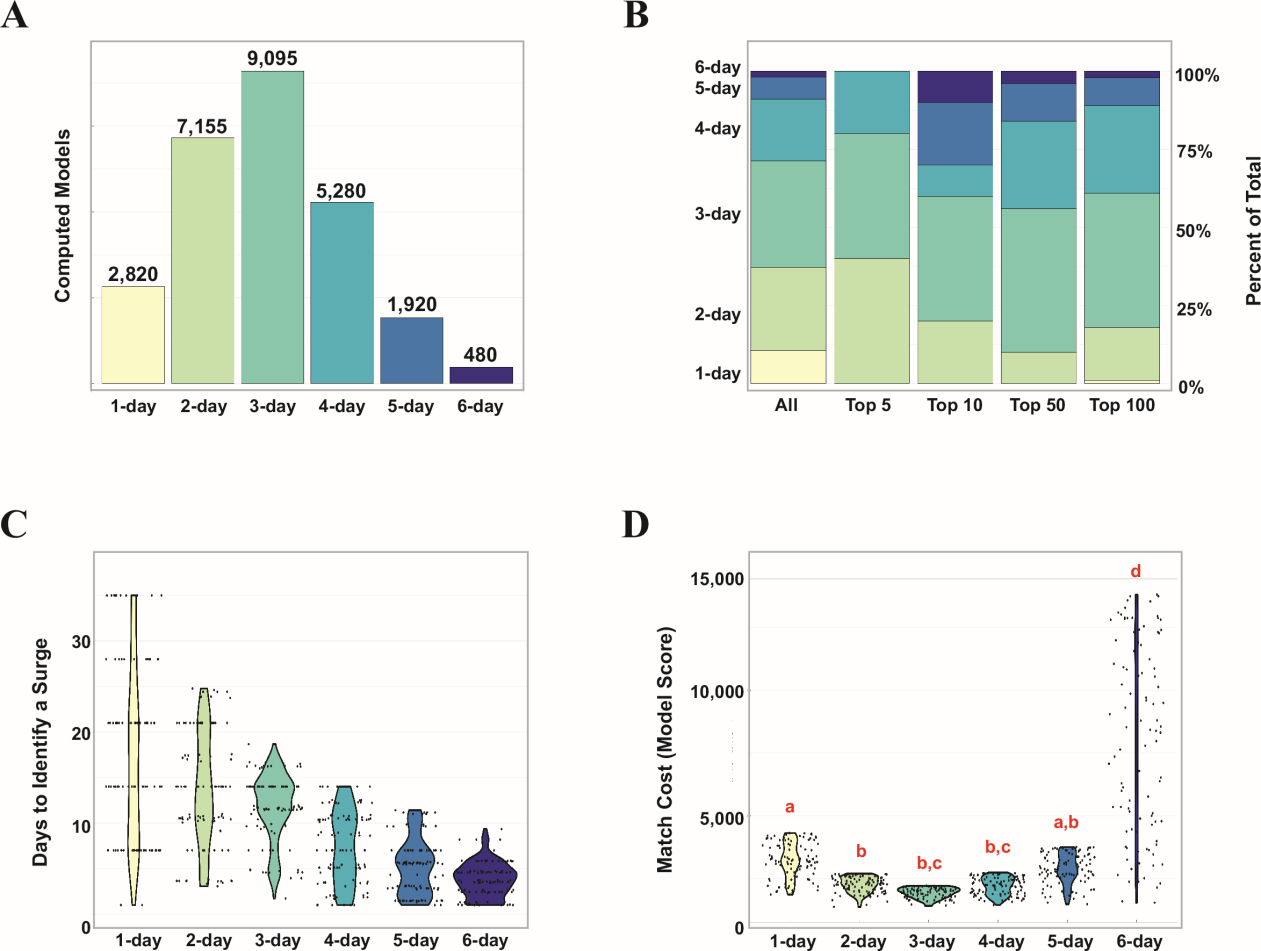
(**A**) Total number of models computed for each collection frequency (1–6 days per week). (**B**) Proportion of models from each collection frequency in all models, the top 5, top 10, top 50, and top 100 scoring models. (**C**) Distribution of the days to identify a surge in the top 100 scoring models from each collection frequency. (**D**) Distribution of the match costs (model scores) among the top 100 scoring models from each collection frequency. Red letters indicate statistical significance (*P* < 0.05) as determined by one-way ANOVA and Tukey HSD analysis in R v 4.3.3.

Model surgesets that used three collections per week (3-day models) overall performed the best in our study. They represented 34% (*n* = 9,095) of all models in the study (Fig. 6A; Table 1) but comprised 60%, 40%, 46%, and 43% of the top 5, 10, 50, and 100 scoring sets, respectively (Fig. 6B; Table 1). The best-scoring 3-day models provided only a slight advantage compared to the best-scoring 2-day models when it came to the time required to identify a surge; however, the best-scoring 3-day models required slightly longer (11.6 days) than the top-scoring model (10.6 days), although the mean of all 3-day models in the top 10 required fewer (8.7 days).

**TABLE 1:** Count of models (n) and percent of all models (%) that comprise the top-scoring 5, 10, 50, and 100 models, as well as all computed models (total), across all values of *f* (collection frequency in days per week) in the current study

*f*	Total (n)	Total (%)	Top 5 (n)	Top 5 (%)	Top 10 (n)	Top 10 (%)	Top 50 (n)	Top 50 (%)	Top 100 (n)	Top 100 (%)
1	2,820	10.5	0	0	0	0	0	0	1	1
2	7,155	26.8	2	40	2	20	5	10	17	17
3	9,095	34.0	2	40	4	40	23	46	43	43
4	5,280	19.7	1	20	1	10	14	28	28	28
5	1,920	7.2	0	0	2	20	6	12	9	9
6	480	1.8	0	0	1	10	2	4	2	2

Models based on two collections per week were also enriched at the very top, representing 26.7% of all computed models but 40% of the top five scoring surgesets (Fig. 6B; Table 1). In addition, the top-scoring model was a 2-day model. However, 2-day models outside of the top five were generally under-represented among the top 10, 50, and 100 scoring sets. Models that used one collection per week were under-represented in all of the top-scoring models, with the best model at this collection frequency falling outside the top 50.

The total number of surgesets varied with collection frequency (*f*), as demonstrated in Fig. 6A, since each value of *f* has a different number of combination of collection days. To control for this, we restricted our analyses for Fig. 6B and Fig. 6C to just the top 100 models for each collection frequency. Results of these analyses indicate that the time required to identify a surge was inversely related to collection frequency, with 6-day models fastest and 1-day models slowest overall (Fig. 6C; Table 2). Model scores were, on average, better for 3-day models than any other frequency (Fig. 6D; Table 2), although scores for models based on 2–5 days were not statistically different from each other.

**TABLE 2:** Mean match cost (model score) and time to identify a surge (*d*) for all scored model surgesets across different collection frequencies (*f*)

*f*	Match cost		Days to identify a surge (*d*)	
	Mean	SD	Mean	SD
1	3,259.6	693.5	17.7	9.7
2	2,274.9	307.4	13.7	6.3
3	1,948.6	199.9	11.8	3.7
4	2,229.9	344.9	7.4	4.0
5	2,920.7	598.4	5.2	3.1
6	8,107.7	3,780.2	3.8	1.9

### Control set does not drive model performance

The control surgesets used to score the models were built from the full data set using the slope of the change in abundance over rolling windows of 2–6 days. To explore the effect of control set on model performance, we analyzed the top 100 scoring models for each control set. Models based on 3-day frequencies dominated the top scorers regardless of the control set (Fig. S2), with distributions similar to that of the full set of models (Fig. 6A).

## DISCUSSION

Previous studies have shown that the abundance of SARS-CoV-2 in wastewater can be used as a useful indicator of clinical case load in a community (14, 24, 25). Similar studies have shown this holds true for other pathogens as well (26, 27). The ability to rapidly and accurately identify trends in wastewater abundance of a pathogen can be a valuable contributor to public health surveillance, enabling agencies to act more proactively than reliance on case data or hospitalizations alone. A challenge to address before it can reach full potential as a public health surveillance tool is the noise inherent in wastewater data. It is a complex and rapidly changeable matrix, subject to a wide array of physicochemical factors that vary geospatially, over time, and longitudinally from source to treatment facility. Additional variability is introduced by the biology of each specific pathogen and its interaction with human hosts, including variable rates of pathogen shedding over time and between individuals; variable retention rates within the sewer systems; variable rates of pathogen degradation; mode of pathogen shedding; and more.

In the current work, we followed the abundance of SARS-CoV-2 by collecting 24-h composite samples from a single treatment facility daily for nearly 4 years. We used this data set to compare different models for accurately identifying surges in pathogen abundance. Our results suggest that collecting two 24-h composites per week is sufficient to identify the start of a wastewater surge within about 10 days. This approach outperformed models that used more frequent sampling and longer windows. Interestingly, the best model scores on average were achieved by 3-day models (Table 2), although they were not statistically different from 2-day and 4-day models at a *P*-value threshold of 0.05. The U-shaped curve of the score vs. frequency plot (Fig. 6D) suggests that there is a "sweet spot" for wastewater collection around 2–4 days per week that balances sensitivity with noise reduction.

We anticipated that more frequent sampling would provide more accurate surge identification, and the failure of higher frequency collection to improve trend identification seems counterintuitive. This could be a result of the specific data set used in this study, suggesting another collection site might exhibit different behavior. Alternatively, it could be a function of the complex nature of the wastewater matrix, as discussed above, such that more frequent sampling actually decreases the signal to noise ratio (28, 29). This may occur as a result of biased (non-random) matrix effects such as temporal heterogeneity. A similar effect was observed, for example, in the relationship between fecal enterococci abundance and the tidal cycle in coastal beach sands of California (30). The effect of sewershed size and structure on wastewater signal is also largely unknown. For example, a significantly larger sewershed than our study site (approximately 50,000 served) is likely to have longer retention times (time it takes pathogens that are shed upstream to arrive at the downstream facility). This could affect the signal strength due to viral particle decay. It could also affect the signal to noise ratio in unpredictable ways, increasing the signal as a consequence of a larger infected population, and/or increasing the noise due to more complex retention dynamics in the system. It is encouraging that our study aligns with previous work using case counts, which suggests robustness, but the broad applicability of our approach remains to be explored. Although long-term, high-frequency wastewater data sets are not common, potentially making these hypotheses difficult to test, our code is freely available (23) and applicable to any wastewater data set.

Out of the top 10 scoring models, 50% (*n* = 5) came from models scored against control set 2 (Fig. 5B), which was constructed from the full abundance data using the rolling slope of two consecutive samples. The significance of this enrichment of control set 2 at the top is not clear. There is no objective way to determine the "true" surges in a wastewater set; hence, there is no way to rank our methods of constructing the control sets. If nothing else, the enrichment of top-scoring models scored against control set 2 suggests good agreement between this control set and our scoring approach. This agreement does not appear to be biased by collection frequency; the distribution of models from different frequencies against the five control sets is similar to each other (Fig. S2) and comparable to the distribution across all models (Fig. 6A), with day-3 models largely dominating the top scorers against each control set. The highest percentages for 2-day, 3-day, and 4-day models did occur in control sets 2, 3, and 4, respectively; however, this pattern did not hold true for 5-day and 6-day models.

It is intriguing to note that collections for the top three scoring models overall were evenly spaced during the week (Fig. 5B)—either Wednesday/Saturday or Monday/Wednesday/Saturday. Although not definitive, it suggests that spacing out wastewater collection may be advantageous for picking up trends. For example, wastewater collected over the weekend may capture recreational activities, while weekday collections capture more work-related activities, and both are required for best model fit. To examine this possibility in more detail, we calculated the mean distance between collection days for each surgeset and then plotted those values against the surgeset match cost (model score) for the top 5, top 10, top 50, and top 100 best-scoring models (Fig. S3). The trend of improving model scores with larger gaps between collection days held true for the top 5 and top 10 best-scoring models overall (Fig. S3A and B), which seems to support our hypothesis that spacing collections out over the week can improve model fit. In contrast, no obvious relationship between gap size and model score was apparent for the top 50 or top 100 models (Fig. S3A and B); however, these model sets had higher overall match costs (i.e., worse scores) compared to the top 5 and top 10 sets. It is possible that the relationship between gap size and model fit diminishes above a certain match cost threshold, although this remains to be tested.

Previous work has demonstrated a strong correlation between COVID case counts and wastewater data collected twice weekly specifically (14). Our study supports this previous work, although it was designed to identify sustained increases in viral wastewater abundance, not measure the interdependence between viral wastewater abundance and case counts; indeed, case counts were not used in any part of the current work. Case counts are an obvious choice for following trends in disease transmission, when they are timely and testing is relatively widespread. In the absence of timely case count data, on the other hand, our wastewater model may be useful in identifying sustained surges in infection levels in a community in time to inform effective public health responses. In resource-limited environments, the current work supports limited sampling (as infrequent as twice weekly) distributed evenly across the week as a useful approach for identifying infectious disease trends within about 10 days. This represents a similar response time compared to provisional case counts, which are released weekly. In urgent situations, collecting three times per week can enable trend identification within just a handful of days with only a slight impact on model score.

## MATERIALS AND METHODS

### Sample collection, processing, and SARS-CoV-2 quantification

Untreated wastewater was collected by facility personnel from the influent pipe (downstream of the grit filter) of a single wastewater treatment facility (population served approximately 50,000) from November 2020 through June 2024. Each individual sample was a 24-hour, flow-based composite of liquid influent collected with the facility’s equipment. Samples were collected 5–7 days per week and stored at 4°C by the facility until retrieval by lab personnel. Samples were picked up weekly, transported to the testing lab on ice, stored at 4°C, and processed within 48 h of receipt; 10 mL of each sample was mixed by inversion, allowed to settle for 10 min, and then concentrated to a final volume of 475 µL using Nanotrap Microbiome A particles (Ceres Nanosciences) on a Kingfisher Apex (Thermo Scientific). Total nucleic acids (TNA) were extracted from 400 µL of each concentrate on the Apex using the MagMax Viral/Pathogen Nucleic Acid Isolation kit (Thermo Fisher Scientific), and eluted in a final volume of 75 µL. Reverse transcriptase droplet digital PCR (RT-ddPCR) was carried out with the One-Step RT-ddPCR Advanced Kit for Probes (Bio-Rad Technologies) on a QX600 ddPCR system (Bio-Rad Technologies) using 5 µL of TNA as input. Total SARS-CoV-2 was measured using the 2019-nCoV CDC ddPCR Triplex Probe Assay (Bio-Rad Technologies), which amplifies the SARS-CoV-2 N1 & N2 targets. The abundance of SARS-CoV-2 in the original sample was calculated from the ddPCR results for the N2 target and reported as the number of target copies per mL of wastewater. All laboratory protocols were carried out under enhanced biosafety level 2 (BSL-2) conditions approved by West Virginia University’s Institutional Biosafety Committee under protocol #20-05-03.

### Modeling surges

#### Modeling the start of a surge

A primary goal of this study was to determine the most effective way to identify surges in the abundance of SARS-CoV-2 in wastewater. To this end, we computationally constructed a set of modeled surges, *M*, by iterating through the sample abundances in *A* in chronological order, using an overlapping rolling window of size *n*. At each step, we calculated the mean of the *n* samples in the window, then compared this mean (*a_i_*) to the previous *s* mean values (*a_i-1_* to *a_i-s_*). If the values from *a_i-s_* to *a_i_* were strictly increasing, we recorded *a_i-s_* as the start of a surge.

#### Modeling the end of a surge

To identify the end of an existing modeled surge, we compared the current value of *a* (*a_i_*) to the previous *t* values of *a* (*a_i-1_* to *a_i-t_*). If the values from *a_i-t_* to *a_i_* were strictly decreasing, we recorded *a_i-t_* as the end of the surge.

Using this approach, we constructed sets of modeled surges *Q* by iterating over all combinations of *n* = [1,6], *s* = [1,4], and *t* = [1,4]. We followed this approach using subsets of the full abundance data set *A* to include all combinations of 1–6 days of collection per calendar week. We hereafter refer to each subset according to the number of collection days per week included in that subset, yielding 1-day models, 2-day models, 3-day models, 4-day models, 5-day models, and 6-day models. (Note: there were no 7-day models; since only eight samples over the entire study period were collected on Fridays, we excluded this day from our analysis.) In the end, *Q* contained a total of 26,750 sets of modeled surges (modeled surgesets).

### Construction of control surgesets

SARS-CoV-2 abundance data from the full set of collections *A* was used to construct a set of five control surgesets, *B*. To start, we iterated over *A* in chronological order and calculated the slope of the line defined by rolling windows of 2–6 consecutive samples. For each window width, we determined two threshold values: one for surge starts based on the most positive slope value, and one for surge ends based on the most negative slope value. This dynamic thresholding approach was implemented because the range of slope values was not consistent but decreased with increasing window width (Table S2). We subsequently iterated over the slope values for each window width in chronological order. If a step had a positive slope that exceeded the start threshold, and it *was not* already part of an existing surge, it was identified as a control surge start. If a step had a negative slope that was lower than the end threshold, and it *was* already part of an existing surge, it was identified as a control surge end. Each surge start and end pair defined a single control surge, and the total set of control surges for a specific window width made up the control surgeset. These five control surgesets (ctl2–ctl6) served as a basis for comparison to score modeled surges as described below.

### Scoring modeled surgesets

We scored each of the 26,750 modeled surgesets in *Q* by comparing it to each of the control surgesets in *B*. Each pairing of a modeled surgeset *q_i_* with a control surgeset *b_j_* was formulated as a bipartite directed acyclic graph, *G*. The “left” side of *G* (*g*_left_) was composed of nodes representing each modeled surge in set *q_i_*. Similarly, the “right” side of *G* (*g*_right_) was composed of nodes representing each control surge in *b_j_*. Each node *m* in *g*_left_ was connected by a directed edge to every node *n* in *g*_right_, making *G* a completely connected bipartite DAG. Each edge in *G* was assigned a positive weight *w_m_,_n_* corresponding to the Euclidean distance in days between the start (*t*) and end (*u*) dates of the modeled and control surges, respectively.

#### Accounting for an unbalanced *G*

Many instances of *G* were unbalanced, wherein the number of nodes in *g*_left_ and *g*_right_ was not the same. To account for an unbalanced *G*, we added “dummy” nodes to the smaller sub-graph until |*g*_left_| = |*g*_right_|. As for non-dummy nodes, each dummy node was connected to all nodes in the companion subgraph; however, each edge that included a dummy node was assigned a weight equal to the largest actual weight on that specific *G* (*w*_max_). This weighting approach was taken in an attempt to balance (but not over-penalize) the cost of modeling too many, or too few, surges compared to the control set.

To assign a score to each modeled surgeset, we calculated a minimum optimal matching for each *G* using the bipartite graph formulation of the Hungarian algorithm (31) as implemented in the RcppHungarian (v 0.3) package of R. The sum of edge weights in this matching was used to calculate a match cost *c* for each modeled surgeset. Note that a lower match cost corresponds to better fit between the model and the control. Finally, we calculated a delay *d* for each modeled surgeset based on how long it required (in calendar days) to identify the surge. This delay was a function of *n, s*, and the collection frequency *f* of the underlying data set. It was also affected by the specific day of the week on which the surge started; consequently, each surge had a specific value for *d*, and *d* for each surgeset was calculated as the mean *d* of its component surges. The calculation of *d* was important as it allowed us to quickly separate out modeled surgesets with very low values of *c,* the match cost (good), but impractically high values of *d*, the delay (bad).

## Data Availability

All data and code to run the models described in this article are freely available in the GitHub repository wwsurges (https://github.com/wvuvectors/wwsurges) (23), under the GNU General Public License version 3.

## SUPPLEMENTAL MATERIAL

Supplemental figures msphere.00652-25-s0001.pdf Fig. S1 to S3.

Table S1 msphere.00652-25-s0002.xlsx Detailed results for all scored surgesets using both start and end date matching.

Table S2 msphere.00652-25-s0003.xlsx Detailed results for all scored surgesets using only start date matching.

ASM does not own the copyrights to Supplemental Material that may be linked to, or accessed through, an article. The authors have granted ASM a non-exclusive, world-wide license to publish the Supplemental Material files. Please contact the corresponding author directly for reuse.
