# Supplementary material for: Rapid identification of COVID wastewater surges in the absence of case data
Source: mSphere. 2026 Apr 2;11(4):e00652-25. doi: 10.1128/msphere.00652-25 (PMC13123706; doi:10.1128/msphere.00652-25)
Supplement: Supplemental figures — Fig. S1 to S3. [file msphere.00652-25-s0001.pdf]

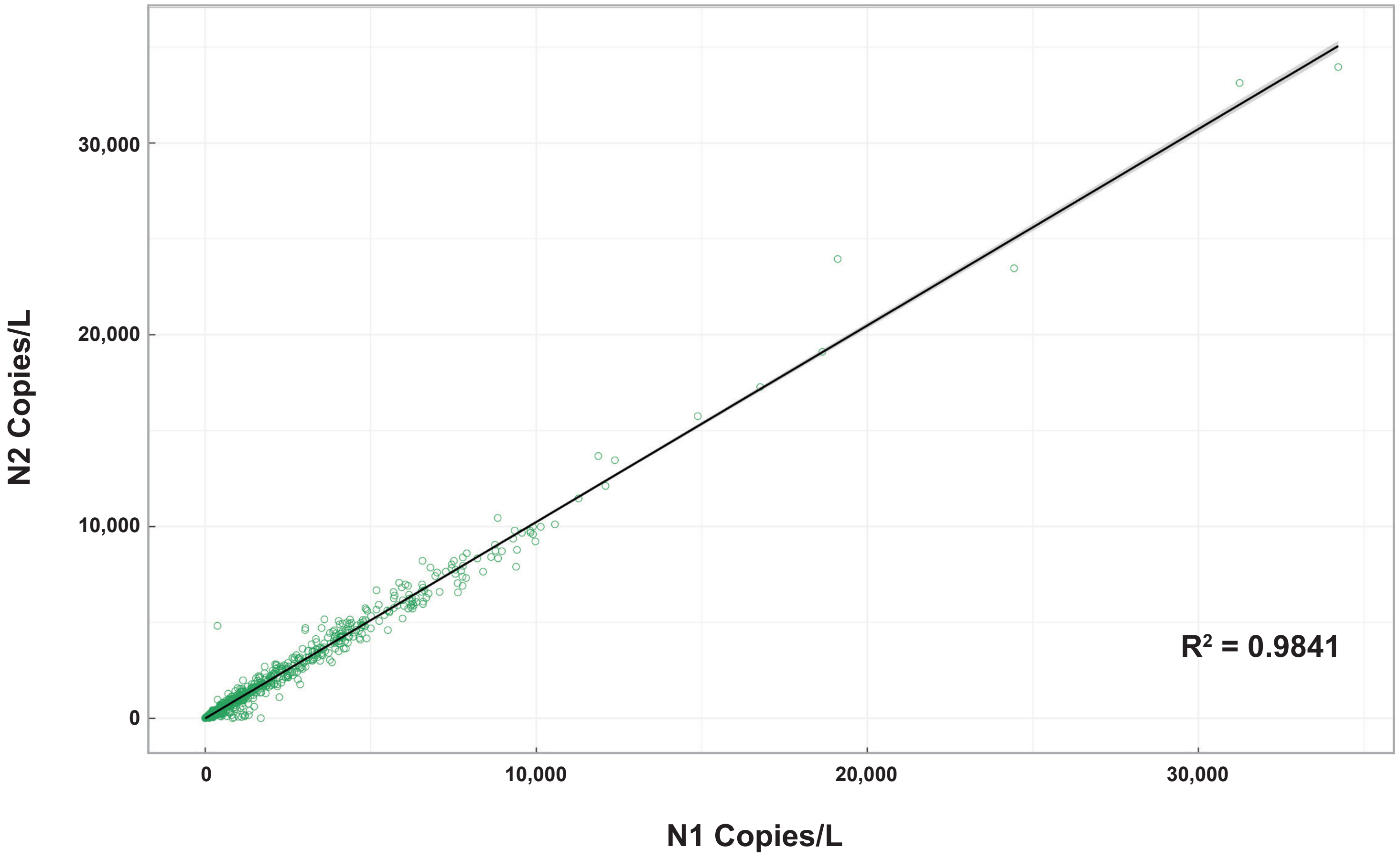

Fig. S1. Correlation between the SARS-CoV-2 N1 and N2 targets in daily abundance data from the study facility. Abundance is reported in copies of the target per liter of wastewater. The black line is the linear correlation as determined by the lm function in R v. 4.3.3. The gray shaded area represents the 95% confidence interval.

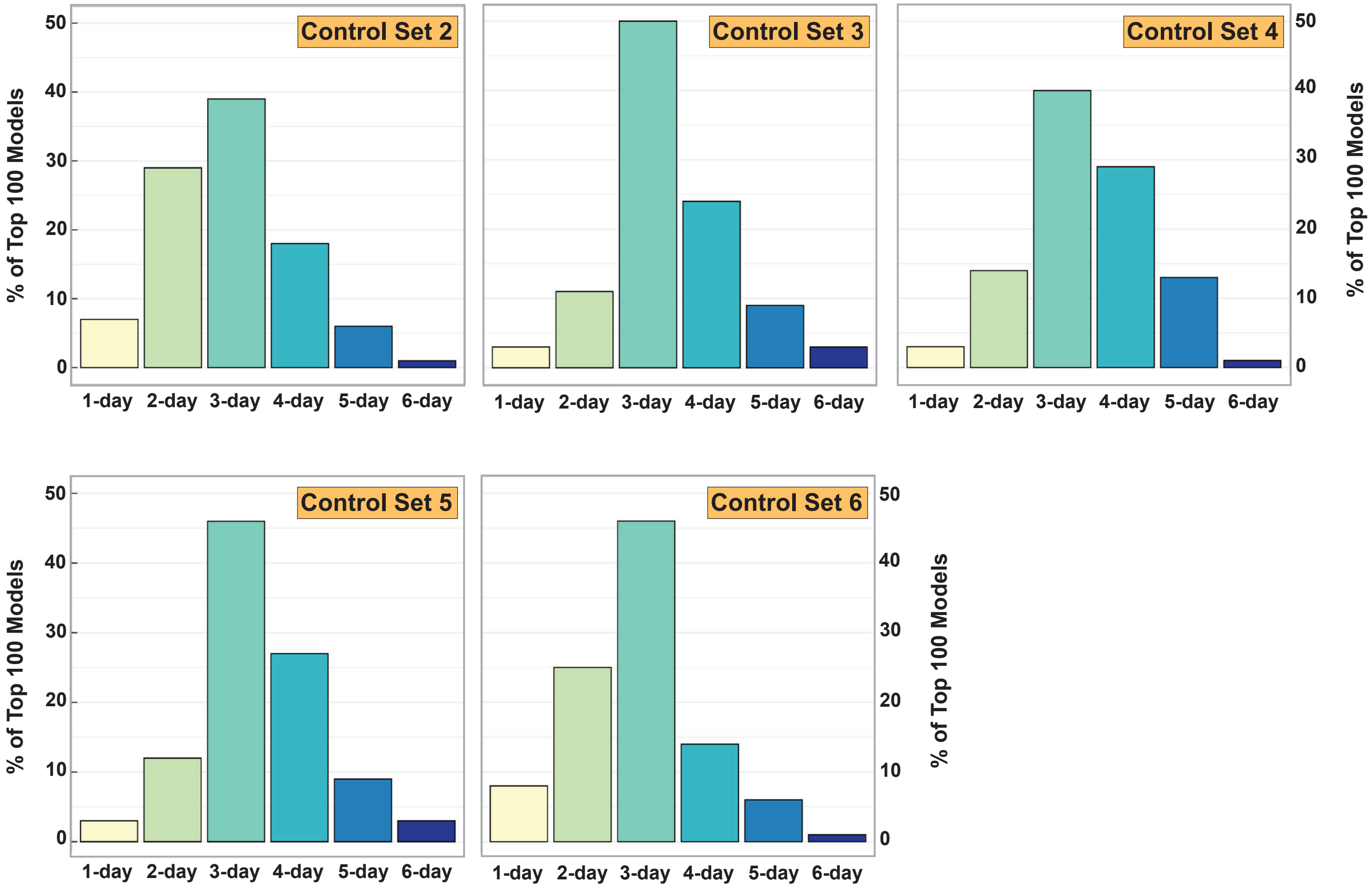

Fig. S2. **Distribution of models from each collection frequency (1 day per week through 6 days per week) in the top 100 models scored against each control set (2-day rolling slope through 6-day rolling slope). See *Methods* in the main text for details on construction of the control sets.**

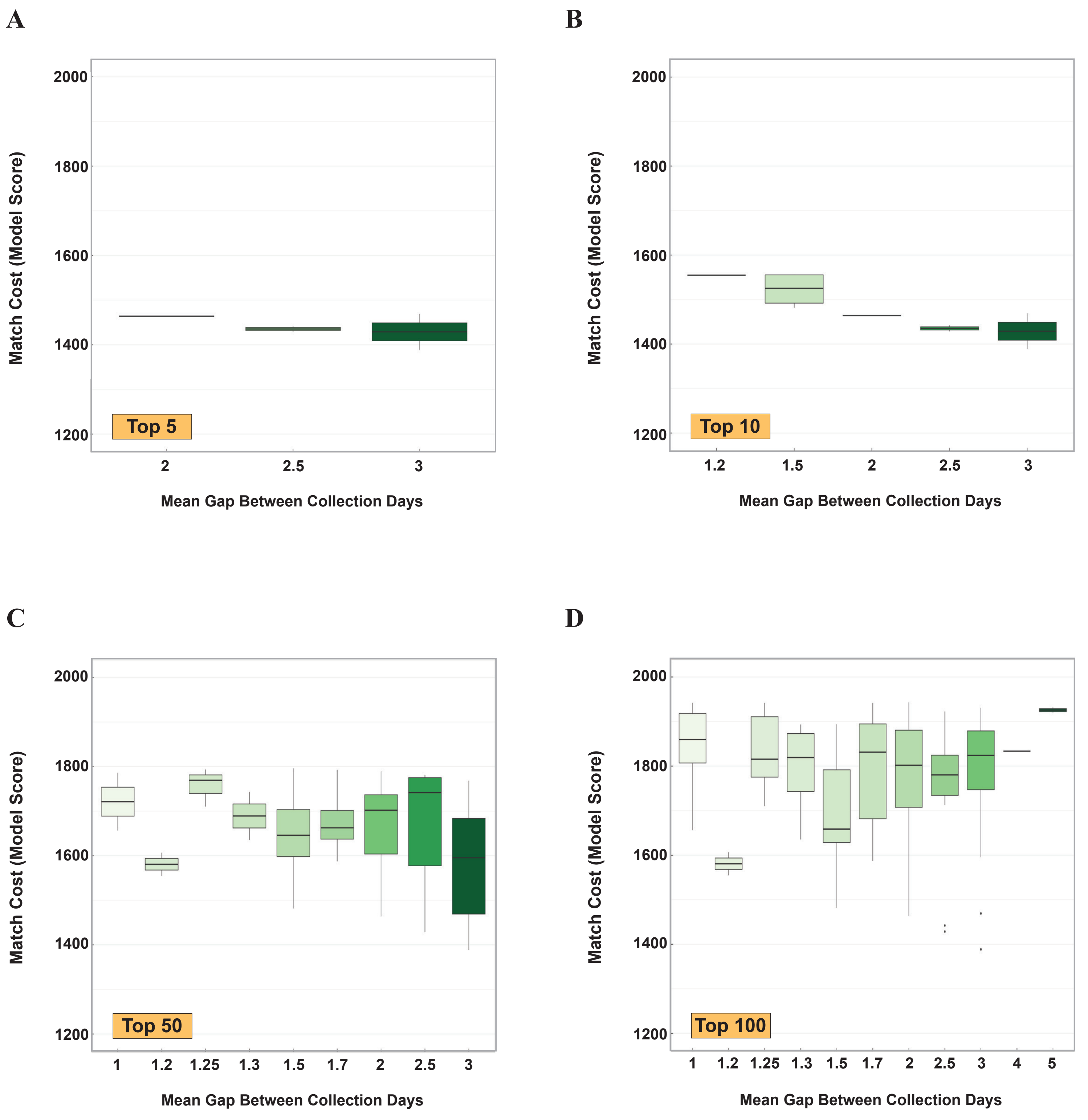

Fig. S3. Mean gap (in days) between collection days for surgesets that comprise the top 5 (A), top 10 (B), top 50 (C), and top 100 (D) scoring models overall. Note that the mean gaps are numerical values but are plotted as categorical data for clarity.
